# The MOBILIZE Boston Study: Design and methods of a prospective cohort study of novel risk factors for falls in an older population

**DOI:** 10.1186/1471-2318-8-16

**Published:** 2008-07-18

**Authors:** Suzanne G Leveille, Douglas P Kiel, Richard N Jones, Anthony Roman, Marian T Hannan, Farzaneh A Sorond, Hyun G Kang, Elizabeth J Samelson, Margaret Gagnon, Marcie Freeman, Lewis A Lipsitz

**Affiliations:** 1 Division of General Medicine and Primary Care, Beth Israel Deaconess Medical Center, Boston, Massachusetts, USA; 2 Harvard Medical School, Boston, Massachusetts, USA; 3 Institute for Aging Research, Hebrew SeniorLife, Boston, Massachusetts, USA; 4 Division of Gerontology, Beth Israel Deaconess Medical Center, Boston, Massachusetts, USA; 5 University of Massachusetts Boston, Center for Survey Research, Boston, Massachusetts, USA; 6 Department of Neurology, Brigham and Women's Hospital, Boston, Massachusetts, USA

## Abstract

**Background:**

Falls are the sixth leading cause of death in elderly people in the U.S. Despite progress in understanding risk factors for falls, many suspected risk factors have not been adequately studied. Putative risk factors for falls such as pain, reductions in cerebral blood flow, somatosensory deficits, and foot disorders are poorly understood, in part because they pose measurement challenges, particularly for large observational studies.

**Methods:**

The MOBILIZE Boston Study (MBS), an NIA-funded Program Project, is a prospective cohort study of a unique set of risk factors for falls in seniors in the Boston area. Using a door-to-door population-based recruitment, we have enrolled 765 persons aged 70 and older. The baseline assessment was conducted in 2 segments: a 3-hour home interview followed within 4 weeks by a 3-hour clinic examination. Measures included pain, cerebral hemodynamics, and foot disorders as well as established fall risk factors. For the falls follow-up, participants return fall calendar postcards to the research center at the end of each month. Reports of falls are followed-up with a telephone interview to assess circumstances and consequences of each fall. A second assessment is performed 18 months following baseline.

**Results:**

Of the 2382 who met all eligibility criteria at the door, 1616 (67.8%) agreed to participate and were referred to the research center for further screening. The primary reason for ineligibility was inability to communicate in English. Results from the first 600 participants showed that participants are largely representative of seniors in the Boston area in terms of age, sex, race and Hispanic ethnicity. The average age of study participants was 77.9 years (s.d. 5.5) and nearly two-thirds were women. The study cohort was 78% white and 17% black. Many participants (39%) reported having fallen at least once in the year before baseline.

**Conclusion:**

Our results demonstrate the feasibility of conducting comprehensive assessments, including rigorous physiologic measurements, in a diverse population of older adults to study non-traditional risk factors for falls and disability. The MBS will provide an important new data resource for examining novel risk factors for falls and mobility problems in the older population.

## Background

Previous research on the causes of falls in older adults has uncovered numerous risk factors, but most epidemiologic studies of falls have focused generally on a common set of factors. Despite the progress in understanding the causes of falls in older persons, not all causes of falls are known and many suspected risk factors have not been adequately studied [1,2]. Nonetheless, multifactorial interventions to prevent falls in older persons have met with moderate success, particularly when a targeted risk factor reduction strategy is employed [3-5]. Because falls are a leading cause of disability in the older population and because factors that contribute to falls are also risk factors for many other adverse consequences in older adults, fall prevention strategies may have the greatest impact overall in reducing disability in the older population [6,7]. As the post-WWII Baby Boom generation approaches old age, there is an urgent need to improve our understanding of the causes of falls and enhance the multifactorial interventions with a more enriched knowledge base.

Selected risk factors for falls such as pain, changes in cerebral blood flow regulation, and foot disorders are poorly understood, in part because they pose measurement challenges, particularly for large observational studies. Assessment of pain in previous cohort studies has generally targeted selected regions such as knee or back pain, or alternatively, summary measures have been used to assess overall pain severity using one or more numeric rating scale items. The few studies that have examined pain location using pain maps and other comprehensive approaches to pain assessment have shown that location of pain throughout the body is an important predictor of falls and disability [8-11].

Although postural blood pressure declines are known to be associated with falls [12,13], it is not clear whether alterations in cerebral blood flow (CBF) regulation may contribute to falls in community-dwelling seniors. There is evidence that orthostatic hypotension and postprandial hypotension are associated with subcortical white matter abnormalities, presumably due to ischemic injury to watershed areas of the brain during periods of hypotension [14]. Increased blood pressure variability is also associated with white matter abnormalities in the brain [15,16]. Furthermore, several case-control studies have demonstrated that ischemic white matter changes are associated with gait and balance abnormalities, which may lead to falls [17-19]. However, to our knowledge, no studies have shown a direct relation between cerebral blood flow abnormalities and falls in representative community-based populations. Cerebral blood flow and its regulation can be measured non-invasively and economically with good temporal resolution using Transcranial Doppler Ultrasonography (TCD). This technique is particularly suitable for population-based studies and offers the potential to better understand the role of CBF abnormalities as a cause of falls, syncope, and impairments in gait and balance.

Foot disorders are often overlooked as important potential causes of falls and little is known about how foot disorders influence important potential mediators of falls, such as balance or gait in older persons. Tinetti et al. found that elders with self-reported 'serious foot problems' were more likely to fall during the 1-year follow-up than those without foot problems [20]. Two retrospective studies found that self-reported foot disorders increased the risk of falling in elderly persons [21,22]. In one of the few prospective studies of foot problems and falls, Menz and colleagues followed 176 elderly Australians for one-year and found that severe hallux valgus deformity, decreased plantar tactile sensitivity, and foot pain were significantly associated with falls [23]. These studies indicate that foot disorders are likely to be linked to falls, however, the definitions of foot problems used in many studies were often limited and based on self-report. No studies to date have examined the relation between foot disorders and falls with empirical measures of balance and footwear, as well as measures of functional performance.

The MOBILIZE Boston Study (MBS), which stands for "Maintenance of Balance, Independent Living, Intellect, and Zest in the Elderly of Boston" is a major new cohort study, part of the Hebrew Rehabilitation Center/Harvard Research Nursing Home Program Project. Each of the projects within the MBS is targeting one of these novel risk factors for falls. The Program Project, funded by the National Institute on Aging, is likely to have a significant impact on our understanding of hazards posed by problems of pain, cerebral hypoperfusion, and foot disorders in the older population. In this paper, we present the design and an initial description of the MBS study cohort recruited from an urban population of older adults.

## Methods

### MBS project overview

The MBS was designed to efficiently address multiple project aims, by using core resources to recruit and study a large population of aged individuals. Furthermore, the identification and careful characterization of a diverse, elderly, community-based population has enabled us to create a valuable database to foster future research beyond the scope of the MBS, including the future study of other lifestyle factors, biomarkers and even genetic factors that may underlie risk for falls and disability in older populations.

### Study design

The MBS is a prospective observational study based in the Institute for Aging Research (IFAR) at Hebrew SeniorLife, a large geriatric housing, health care, and research organization. Study operations are centralized in the Institute where the staff coordinates all aspects of participant enrollment, data collection and management, and participant follow-up. The MBS is a collaborative effort involving investigators at IFAR, Beth Israel Deaconess Medical Center, Harvard Medical School, the University of Massachusetts Boston (UMASS), and Boston University. The population-based recruitment was conducted by the UMASS Center for Survey Research (CSR) in close collaboration with IFAR's outreach staff.

Once recruited through home visits, elders were contacted by telephone by IFAR research staff to confirm eligibility and schedule the 2-part baseline data collection that comprised a home visit and an examination at the MBS research clinic based at IFAR. During the home visit, participants were given a set of monthly falls calendar postcards to be completed and mailed to IFAR at the end of each month. In the ongoing follow-up, a telephone interview is conducted whenever a fall is reported on the monthly calendar. The data collection is repeated 18 months following enrollment, and uses the same 2-visit approach. The MBS was approved by the Institutional Review Boards of Hebrew SeniorLife and the collaborating institutions.

Within the program project, subgroups of the population-based cohort are being invited to participate in clinical projects that address specific questions related to the mechanisms and prevention of falls. One of these investigates the effect of subsensory mechanical "noise" applied to the soles of the feet through a vibrating sandal on balance and gait [24-26]. This project examines healthy elderly fallers and non-fallers who have intact sensation in the feet, and those with peripheral neuropathy. A second project examines the anatomical and cerebrovascular regulatory changes of the brain associated with slow gait speed using MRI and TCD. Subjects with a gait speed of < 0.6 m/sec during the 4-meter walk and controls matched on age, gender and cardiovascular risk factors are targeted for this study.

### Participant recruitment

As of January, 2008, the MBS recruitment was concluded and 765 participants completed the 2-part baseline assessment. The recruitment strategy targeted older persons aged 70 years and older, living within a 5-mile radius of IFAR by using a simple random sample of persons on the town lists. Since the lists included approximately 90% of all people 70 or older in the area, all of these people had known probabilities of selection. A comparison of the demographics of persons on the town lists with the US Census 2000 showed that the town lists have a comparable distribution by age and sex in the age 70 and older population.

The geographic boundary, chosen to facilitate recruitment and limit transportation burden and costs, included a wide variety of neighborhoods in Boston and surrounds ranging from ethnically and socioeconomically diverse urban communities to suburban regions with predominately white, middle-class residents. According to the U.S. Census 2000, among persons aged 70 and older in this locale, the minority representation was approximately 19%, which was lower than the general Boston population across all ages. Extra efforts were made to recruit in areas with large minority representation, but oversampling was not needed.

In preparation for recruitment, letters were sent to local police departments, fire stations, and churches informing them about the study, and informational newspaper articles and flyers were distributed to residents. Randomly selected households in the target area were sent letters informing them that a research assistant from the CSR would be visiting their home to discuss the study. During their first contact with potential subjects, staff explained the study and performed a rapid eligibility screen. Eligibility criteria included age 70 years or older, ability to speak and understand English, ability to walk across a small room, sufficient vision to read written material, and the expectation that they will be living in the area for at least 2 years. Companions or spouses who were aged 65 or older living with a participant also were allowed to join the study, as it was recognized early on that recruitment of one spouse or companion without the other would limit participation. Study participation was limited to English speakers because it was not feasible to translate the study instruments and conduct the interviews in the many languages that are spoken within Boston's minority communities.

### Subject screening and recruitment

Persons who expressed interest in participating after being contacted at home by the CSR were referred to IFAR research staff who conducted the initial screen by telephone. Eligibility criteria are shown in Table 1. Final eligibility was determined at the start of the home interview, including ascertainment of ability to walk across a small room without personal assistance and a screen for cognitive impairment. We excluded persons with a Mini-Mental State Exam (MMSE) score less than 18, indicative of moderate or severe cognitive impairment [27,28]. In addition, at the home interview, the research assistant confirmed that there were neither serious language difficulties nor severe visual or hearing deficits. Severe sensory deficits and moderate or severe cognitive impairment precluded the participants' ability to perform the various tasks and procedures that were part of the MBS protocol, including informed consent, cognitive and physical performance testing, and monthly falls calendars. Persons who scored 18 or greater on the MMSE but exhibited cognitive difficulties were eligible as long as they demonstrated that they could comply with the study requirements at baseline.

**Table 1:** Study eligibility criteria for the MOBILIZE Boston Study

**Inclusion criteria**
Age ≥ 70 years (or age ≥ 65 if living with an MBS participant)
Able to understand and communicate in English
Plans to be in area for 2 years
Able to walk 20 feet without personal assistance (walking aids permitted)
**Exclusion criteria**
Terminal disease
Severe vision or hearing deficits
Cognitive impairment (Mini-Mental State Examination < 18)[27,28]

### Data collection

The in-home interview and clinic exams were designed to collect extensive information to meet the aims of the projects that comprise the MBS, each of which examines a set of novel risk factors for falls. For multivariable analyses, we also have collected a standard set of established fall risk factors and potential confounders, carefully selected to obtain optimal measures while also keeping subject burden to a minimum in an already lengthy data collection. During the baseline home visit, the interviewer obtained informed consent from the participant, reviewed the study procedures including instructions for completing the monthly fall calendars, and conducted the baseline interview, generally requiring 3 hours. The in-clinic appointment, conducted by research nurses, also lasted approximately 3 hours and took place within 4 weeks of the in-home visit. Measures obtained in the 2-part baseline assessment are described in more detail below, and summarized in Table 2. Participants were given $15.00 for each in-home visit and $30.00 for the in-clinic appointment. Transportation to the MBS clinic using commercial transport vans was provided for all participants as needed or requested.

**Table 2:** Summary of data collection and equipment.

**Domain**	**In-home interview:**	
Chronic conditions	Medical history	
Pain	Pain location & characteristics (McGill Pain Map, BPI)	
Falls	Falls, fractures, and syncope history	
Cognition	Mini-Mental State Exam; Neuropsychological Battery: HVLT-R, Clock-in-the-Box, Verbal fluency, Trails A & B	
Depression	Depression (CESD-R)	
Medications	Medication Inventory (prescription and OTCs)	
	Self-efficacy for self-management (CPSS; PEPPI)	
Footwear	Footwear/shoewear	
Demographics	Socio-demographic information	
Behaviors	Smoking, alcohol use, physical activity (PASE)	
Environment	Perceived neighborhood walkability; Observational environmental assessment	

	**Clinic Exam**	**Equipment**

	Height and weight measured	Stadiometer; balance scale
Mobility performance	Physical performance tests (SPPB)	Stopwatch
Muscle strength	Leg muscle strength (1 RM) and muscle power	Keiser Leg Press
Balance	Standing balance +/- divided task, Berg balance, center of pressure and sway	Stop watch; Kistler force plate
Cerebral blood flow	Transcranial doppler measures	Doppler ultrasonography
Musculoskeletal	Musculoskeletal exam; manual tender point exam	Goniometers, inclinometer
Peripheral neuropathy	Modified Semmes Weinstein neuropathy assessment	Monofilaments
Foot disorders	Foot exam, foot pressure tests	MatScan Foot pressure mat, tuning fork
Blood specimens	Non-fasting blood tests & DNA banking	Phlebotomy equipment
Vision	Vision exam	Good-Lite Chart™

### Baseline home interview

The baseline home interview, conducted by trained research assistants, included extensive information about health and functioning: chronic diseases (self-report of physician diagnosis and the Rose Angina and Claudication Questionnaires [29]), health behaviors (smoking, alcohol use, walking activity [30]), self-efficacy for pain and disease management [31,32], social network and support [33], pain assessment (described below), fall history, fracture history, medication adherence [34], and sociodemographic characteristics. Three domains of disability were assessed, Activities of Daily Living (ADL: bathing, dressing, transferring, using the toilet, and eating [35]), Instrumental Activities of Daily Living [36] (IADL: shopping, preparing meals, and housework), and lower extremity mobility (walking and stair-climbing) [37]. Response options for the ADL, IADL, and mobility items included asking individuals to identify their level of difficulty (none, a little, some, or a lot) or inability in performing each ADL and IADL activity. At the conclusion of the home visit, the interviewer conducted a brief observational assessment of the home environment to assess for fall hazards, such as obstacles on the floor and condition of stairways inside and outside the home, and presence of adaptive equipment, such as grab bars in the bathroom.

#### Cognitive tests

Verbal memory functioning was assessed with the Hopkins Verbal Learning Test – Revised (HVLT-R). The HVLT-R is a 12-item word list learning test that has been identified as an ideal memory measure for elderly patients and those suspected of dementia [38]. Verbal fluency was assessed with phonemic and semantic fluency tasks [39]. Reliability and validity of the HVLT-R have been shown in both older adults and persons with frontal lesions [40-42]. The Trailmaking Test (parts A and B), requires the individual to connect encircled items in sequential order in a timed test. The Trailmaking Test, a measure of executive function, is frequently used in the clinical setting and has been shown to be sensitive to the presence of frontal lobe pathology and increased cerebrovascular risk [43]. The Clock-in-a-Box Test (CIB), a modification of the commonly used Clock Drawing test [44,45], was designed as a cognitive screening measure for use in the medical setting and has increasingly been used as a measure of executive function [46].

#### Medications

A medication review performed during the in-home visit included an examination of all containers of prescription and over-the-counter medicines used in the previous 2 weeks, and recording of name, strength and number taken per day, week or month [47].

#### Self-administered questionnaire

At the end of the home interview, participants were given a questionnaire to complete and bring with them to the clinic visit. This self-administered instrument included a well validated measure of social networks [33], the anxiety subscale of the Hospital Anxiety and Depression Scale [48,49], the Physical Activity Scale for the Elderly (PASE) [50], and the Short Form-12 to measure self-rated health, bodily pain, limitations in social and physical activities, and emotional health [51].

#### Pain assessment

We used several measures to assess pain location, intensity and characteristics during the health interview. Participants were asked to identify sites of current pain that lasted more than a week or two, using the McGill Pain Map, a homunculus showing the front and back of a human figure [52]. The method was developed and validated for use in studies of older populations [53]. The multidimensional Brief Pain Inventory (BPI) consists of subscales for pain descriptors, pain-related quality of life, and pain relief [54]. The BPI and its subscales are well-tested and reliable instruments in patients with back pain, arthritis, and peripheral neuropathy [55-57]. Pain severity was measured using the 4-item BPI scale measuring pain intensity in the past week using a 0–10 numeric rating scale, where 0 is no pain and 10 is "severe or excruciating pain, as bad as you can imagine". The 7-item BPI pain interference scale measures level of pain interference with general activity, mood, walking, normal work including housework, relations with other people, sleep, and enjoyment of life. Response levels on the numeric rating scale ranged from 0 (not at all interferes) to 10 (completely interferes). Of note, reports from research staff indicated that participants had little or no difficulty using the BPI's rating scales. To assess nonconventional pain management, we added several additional items to the BPI nonpharmacologic treatment assessment.

A second measure of pain location and severity was a modification of the pain assessment used in the Women's Health and Aging Study (WHAS) [58]. The series of items on sites of chronic musculoskeletal pain addressed presence and severity of back and joint pain (feet, knees, hips, shoulders, hands/wrists). Participants were asked to rate back or joint pain using the same numeric rating scale used in the BPI, described above. The questions were changed to match the American Pain Society's definition of chronic pain as pain lasting 3 or more months, rather than 1 month in the previous year [58]. The WHAS pain measures have been shown to predict falls and disability in older women [8,9].

#### Falls history

Standard questions regarding falls to the ground or lower surface occurring in the last year were used to ascertain fall history [59]. The Tinetti Falls Efficacy Scale (FES) is a 10-item instrument assessing degree of self-confidence in performing daily activities, such as carrying heavy objects, bathing and housekeeping, without falling. The FES has been found to be associated with falls and disability in older adults [60,61].

#### Depression

Depression symptomatology was measured using a modification of the 20-item Centers for Epidemiologic Studies Depression (CESD) scale [62]. The instrument has been shown to be valid, reliable and sensitive to change in older populations [63,64]. Recently, Eaton and colleagues at the Johns Hopkins University developed a revision of the CES-D, adding symptoms and a response option that together satisfy symptom and duration criteria for DSM-IV Major Depression [65]. In the MBS, we used a modification of the Hopkins Revision of the CES-D (CESD-R). We calculated depressive syndrome burden scores using item response theory [66,67] and the metric was set relative to the mean and variance of the MBS sample aged 70–74 years at baseline interview using a mean of 50, standard deviation of 10. To classify minor and major depression, we applied a diagnostic algorithm following DSM-IV. Persons with minor or major depression had to have either anhedonia or dysphoria. Persons with minor depression had a total of two of nine symptom clusters (dysphoria, anhedonia, appetite disturbance, sleep disturbance, difficulty thinking, guilt, fatigue, psychomotor retardation, or suicidal ideation), major depression requires five of nine symptom clusters. Symptoms within clusters had to be present nearly every day for two weeks in the previous month to meet duration criteria. In an initial sample of 600 MBS participants, the items that comprise the CESD-R were highly internally consistent (coefficient alpha = 0.87).

#### Footwear

We collected information on typical footwear as worn currently and also historically (at ages 20–29, 30–44, 45–64, 65–74 and 75+ years), using a checklist derived in two other population-based studies [68,69]. The focus of the assessment was to distinguish types of typical shoe wear that constrict or place strain on the foot (e.g., narrow toe boxes, elevated heels, absent fixation, excessively flexible heel counters or soles).

### Baseline clinic examination

The baseline examination at the IFAR clinical research center was conducted by experienced research nurses trained in the administration of the complex battery of clinical and performance measures. The intensive assessment was carefully paced to allow rest periods, avoid rushing and prevent excessive burden to participants.

#### Musculoskeletal examination

The primary purpose of the musculoskeletal exam was to assess the American College of Rheumatology's clinical criteria for hip, knee, and hand osteoarthritis, and fibromyalgia, for subsequent adjudication by the study rheumatologist [70-72]. The assessment included observation and movement of hands, wrists, hips, knees, and feet for joint tenderness and swelling, and pain on movement. In addition, we evaluated hip and knee range of motion using a goniometer and inclinometer and assessed for knee angular deformities. The manual tender point exam of 18 tender points assessed criteria for fibromyalgia [73]. Staff were trained by physician specialists (rheumatologist and physiatrist) and certified by demonstrating proficiency in conducting the assessments. An initial reliability study was conducted using 20 elderly volunteers, respectively, to assess inter-rater reliability of the musculoskeletal and tender point exams, and to determine areas of the exams that required further staff training and clarifications to the protocols. Following the additional training, a second reliability study was conducted using 29 volunteers; all measures generally showed good to very good agreement, with kappa statistics ranging from 0.40 to 0.76. Staff have been recertified annually by the trainers in each of the musculoskeletal exam measures.

#### Foot and shoewear assessment

Foot disorders and foot symptoms were assessed using the validated Foot Assessment Clinical Tool to capture the main features of 25 common clinical foot disorders. This instrument has been found to have excellent reliability in 2 cohorts, and validity tested against podiatry examination [74,75]. The Foot questionnaire and examination has two components: the first part queries respondents about pain and specific location of foot pain over various time frames, while the second component consists of a physical examination of the participant's feet. The foot pain questions are useful for global measures and the specific location of pain is used in the identification of specific foot pathology (e.g., plantar fasciitis). Common foot disorders that are examined include Structural Disorders (hallux valgus, pes planus, hammer toes, claw toes, overlapping toes, valgus/varus, Tailor's bunion, amputated toes, other foot deformity), Skin or Nail Disorders (hyperkeratosis, maceration, fissuring, tinea pedis, foot ulcer, ingrown toenail, nail disorders), Systemic Disorders (vascular insufficiency, ankle edema, hallux rigidus, foot infection, fat pad atrophy), and Sensory/Pain Disorders (foot vibratory sensation, Morton's Neuroma, plantar fasciitis, heel spur, local foot pain symptoms). During the Foot Examination, we also collected foot imprint data using a MatScan, a computer-driven foot pressure and imprint device (Tekscan, Boston, MA). This device provides timely measures of foot pressure concentrations, dynamic weight transfer and evaluation of foot function as a participant walks across a sensory mat. The data from this device provide information on foot disorders, such as valgus or varus foot and pes planus during static stance as well as a dynamic walk.

#### Somatosensory function tests

We used an abbreviated Semmes-Weinstein monofilament test (SWMT) to assess the threshold for light touch pressure using a buckling column, which imparts a known force to the skin on the dorsum of each great toe [76]. Touch sensation threshold was measured as a function of column size/buckling force using 2 monofilaments (sizes 4.17 and 5.07). The test results were categorized into sensory loss groupings of mild, moderate, and severe deficits. We also used a common clinical test, the brush test, applying a light touch cotton ball to the soles of the feet to test for hyperalgesia among participants who were found to have abnormal findings on the SWMT.

#### Cerebral blood flow (CBF) regulation

CBF velocity was measured continuously in the middle cerebral artery using transcranial Doppler ultrasonography (TCD) while sitting in a chair [77,78]. A 2 MHz pulsed flat transcranial Doppler probe (MultiDop, DWL) was placed over the right or left temporal bone with the best signal, and held in place during recordings using a Velcro headband. Continuous arterial blood pressure (BP) measurements were obtained simultaneously using a Finometer photoplethysmographic system (Finapres Medical Systems, Arnhem, The Netherlands) on a finger and held at heart level with a sling. After baseline CBF and BP measurements were obtained, the CBF responses to posture change and CO_2 _inhalation and cognitive activation were evaluated. For posture change, a sit-to-stand maneuver was performed [79]. Subjects sat with their legs elevated at 90 degrees in front of them on a stool. Measurements were obtained continuously during a 5-minute rest in the sitting position then while standing upright for 1 minute. The initiation of standing was timed from the moment both feet touched the floor. The response to CO_2 _was assessed using the CO_2 _rebreathing and hyperventilation method. Subjects were asked to inspire a gas mixture of 8% CO_2_, 21% O_2_, and balance nitrogen for 2 minutes and then mildly hyperventilate to an end-tidal CO_2 _of approximately 25 mmHg for 2 minutes. Postural blood pressure (BP) measurements were obtained according to a standardized measurement technique [80]. In a substudy of subjects with slow gait speed and controls, cerebral blood flow changes in response to cognitive activation were measured during a separate visit, as reported elsewhere [81].

#### Berg Balance Scale

The Berg Balance Scale is a multi-component assessment of standing balance, consisting of 14 balance tasks with each task scored from 0 to 4, for a summed score of 0 to 56 [82]. The scale has been well-validated and shown to predict risk of falls in community-dwelling elders [83]. The unipedal stance, also a validated measure of standing balance and risk for falls, is part of the Berg Balance Scale [84].

#### Quiet standing balance and dual task

Subjects were asked to stand on a Kistler force platform (Kistler Instrument Corp., Amherst, NY) to measure postural sway as the displacement of the center of pressure under their feet. Ten, 30-second quiet-standing trials were performed with each participant, half of which include a cognitive task (dual task challenge), randomized to the first or second half by computer. We chose this approach because we felt the protocol would be too confusing for participants and prone to carryover effects if we randomized each trial. Thus, either the first or last five of the ten balance trials included a cognitive task while the individual stood on the balance platform. The cognitive task was serial subtractions, described below. Rest breaks were provided as needed.

We sought to use a "dual task" that: 1) would load attention, 2) would reflect, to some degree, a familiar activity, 3) would not directly influence balance, and 4) would be fairly independent of educational background. We chose, therefore, a paradigm used widely in neuropsychological testing: "serial subtractions." The subject was asked to subtract 3 from 500, when they said the answer, they subtracted 3 again, continuing until they reached the end of the trial. In subsequent dual task trials they continued the subtractions where they previously left off. Performance on the "dual task" was monitored by asking the subject to state the answers orally. If subjects were unable to perform subtractions by 3, the test was modified by having them count backward by 1 from 500, or count backward by 1 from 100, or identify items at a supermarket. The static balance measure was a major outcome for the project on foot disorders. Because of the subject burden associated with the assessments required for the 4 projects, we could not include additional balance measures such as dynamic balance tests that were not central to any of the projects' aims.

#### Vision

Distant vision was assessed using the Good-Lite Chart Model 600A light box. The letter chart used with the light box was designed for use at a 10-foot text distance [85]. For the test, participants read from 9 rows of progressively smaller letters, with each line assigned a score of 10. Their total score was a sum of the successfully identified letters.

#### Physical performance

The Short Physical Performance Battery (SPPB) was used to measure lower extremity mobility performance [86]. The SPPB includes measures of standing balance, 4-meter usual-paced walking speed, and ability and time to rise from a chair 5 times. The validity of this scale has been demonstrated by showing a gradient of risk for admission to a nursing home and mortality along the full range of the scale from 0–12 [87,88]. Leg strength and muscle power was measured using a double leg press (Keiser Pneumatic Leg Press, Fresno, CA). Participants performed 8 to 12 repetitions to determine the maximal leg muscle strength, referred to as 1 repetition maximum (1RM). Leg muscle power was then performed at 40% 1RM (low resistance). The highest of 5 repetitions was recorded as the maximal double leg press power. For the testing, participants were instructed to use the Borg Scale to rate their perceived exertion and to determine their need for rests between repetitions [89].

#### Laboratory measures

Baseline laboratory tests included hemoglobin, hematocrit, hemoglobin A1C, lipid panel and random glucose level. In addition, blood was stored for later evaluation of potential biomarkers and DNA was extracted and stored for future genetic analyses.

### Falls ascertainment

A fall is defined as unintentionally coming to rest on the ground or other lower level not as a result of a major intrinsic event (e.g. myocardial infarction or stroke) or an overwhelming external hazard (e.g. hit by a vehicle) [90]. During the baseline home visit, participants were instructed about how to complete the monthly falls calendar on a postage-paid folding postcard and return it to the study center at the end of each month during follow-up. This validated method has been used successfully in longitudinal studies of falls [7,59,91,92]. Calendar postcards have been used as a gold standard in studies evaluating fall recall at 3, 6 and 12 months in elder cohorts [59,93]. Based on the approach described by Tinetti and colleagues, participants were instructed to mark an "F" on the days that a fall occurred and an "N" for each day that no fall occurred [92]. In addition to the calendar, the monthly postcard included 4 questions. Two items from the SF-12 addressed self-rated mobility difficulty and bodily pain. The latter correlates well with visual analog scales for rating pain [94,95]. Additional items assessed usual footwear during the calendar month, ER visits and overnight stays in a hospital.

During the follow-up, participants who do not complete the calendar or fail to return it within 10 days of the end of the month are contacted by telephone by study staff to determine whether a fall occurred in the previous month. With any participant who reports a fall, a research assistant conducts a structured telephone interview to determine the circumstances and location of the fall, injuries sustained, and the presence of external and internal factors that may have contributed to the fall. Using a detailed algorithm, falls are categorized as follows: nonsyncopal falls, syncopal falls (associated with loss of consciousness), falls due to an overwhelming external hazard, and falls caused by major medical events other than syncope (e.g. stroke, seizure).

### Study follow-up

The 2-part assessment including the home interview and the clinic exam is repeated 18 months following baseline. Thus far, 87% of persons seen at baseline who are due for their 18 month follow-up remain in the study (10% have withdrawn or dropped out and 3% died in the first 27 months of the study), and of those, 98% have completed at least partial follow-up.

### Statistical analysis

Baseline data were available for the first 600 participants enrolled in the study. Baseline demographic and health characteristics were presented using descriptive statistics, primarily frequency distributions and percentages. In the planned prospective analyses for risk for falls and recurrent falls (not presented in this paper), we will employ survival analysis methodology. Although it is beyond the scope of this paper to present every planned analytic approach in the MBS, here we describe a standard methodology that will be used in some of our research studies. Occurrence of falls will be determined based on the monthly fall and event calendars collected during the follow-up. New and recurrent falls will be identified and incident and cumulative fall rates will be calculated according to baseline factors of interest. We will analyze time to first fall (incident falls) using survival analysis methods. Relative risk for incident falls will be estimated from age-adjusted and multivariate-adjusted models, performed using Cox proportional hazards methods with random effects to account for possible dependence of individuals within the same census blocks [96,97]. For the incident falls analysis, a participant will be censored at the time of her/his first fall during follow-up and no later follow-up data from this person will be used in the models. To examine risk for recurrent falls during follow-up (2 or more falls in 12 months), we will use general linear models appropriate for repeatedly observed ordinal outcomes with correlated responses, such as Poisson regression and negative binomial models. In other analyses, we will use structural equation modeling to determine if latent variables defined by shared covariation among groups of potential risk factors contribute to fall risks.

## Results

### Recruitment

**Figure 1:**
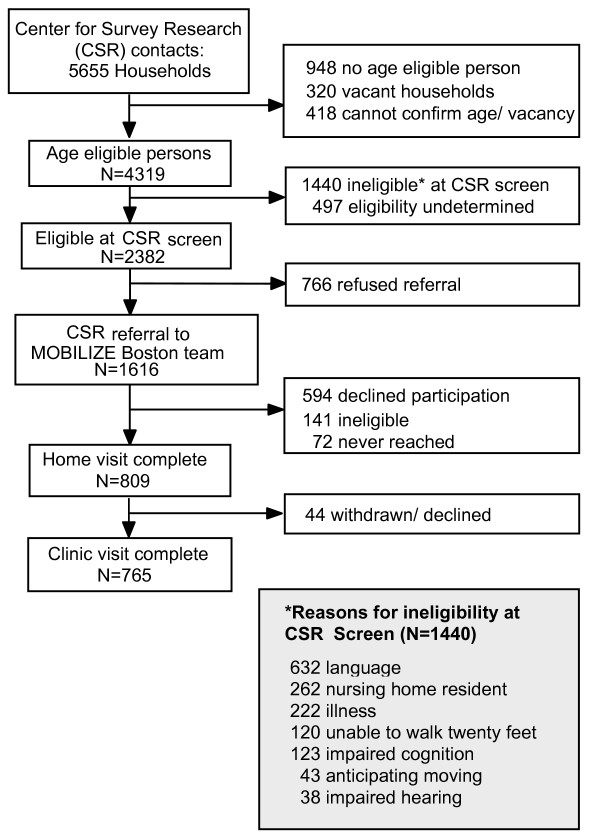
Recruitment and enrollment flowchart, MOBILIZE Boston Study.

The recruitment and enrollment of the 765 study participants are summarized in Figure 1. Among the 5655 households selected for recruitment by the CSR, there were 4,319 people aged 70 and older identified. In total, 88.5% (3822/4319) were successfully screened as to whether they met all study eligibility criteria. Of these, 2382 were eligible and 1440 were not. The primary reasons for ineligibility were language other than English and residing in a nursing home. Of the 2382 who met all eligibility criteria at the door, 1616 (67.8%) agreed to participate and were referred to IFAR for further screening.

When we compared those who were referred to IFAR to all the people who were initially contacted, the age, gender, and race distributions were almost identical (Table 3). We found that persons who were ineligible at the door tended to be somewhat older than the referrals and also compared to the general population over age 70 in the recruitment region as determined by the US Census 200 information. This would be expected as many exclusion criteria are more common among the oldest people. Also, people listing their race as "other" were also ineligible at a higher rate. This was predominantly due to the English language requirement and most Hispanics classified themselves as "other" for race. Therefore the study population closely mirrors the 70 and older population of the sample area once eligibility effects are taken into account.

**Table 3:** Demographic characteristics of the recruitment region, contacted age-eligible persons, referrals to the IFAR study center, persons who were ineligible or refused to participate, and the MBS study enrollees.

Characteristics		2000 U.S. Census*	People encountered at the door	Referred to IFAR	Ineligible at door	Refused at door	Study enrollees (n = 600)^§^
		
		percent					
Age (years):^†^	65–69	-------	0.96	1.63	--------	--------	--------
	70–74	32.41	27.99	27.51	28.31	29.09	31.11
	75–79	27.93	27.55	30.47	22.75	24.28	31.45
	80–84	19.60	22.93	23.49	20.95	23.51	23.76
	85+	20.05	20.57	16.90	27.99	23.12	13.68

Race:	White	80.09	76.89	77.95	69.81	79.11	77.83
	Black	11.93	17.71	17.49	18.63	17.64	16.50
	Other	7.88	5.40	4.56	11.56	3.25	5.50

Gender:	Male	36.36	35.62	36.50	34.04	34.49	35.83
	Female	63.64	64.38	63.50	65.96	65.51	64.17

* Metropolitan Statistical Area of Boston, US Census 2000.

§Data were available only for the first 600 persons enrolling in the MBS cohort. One person was missing race information.

† Age eligibility was 70 years and older except for spouses of participants, who were eligible if within 6 months of their 65^th ^birthday or older.

### Baseline characteristics

The average age of the first 600 study participants was 77.9 years (s.d. 5.5) and nearly two-thirds were women. The study cohort was 78% white and 17% black (Table 3). In comparison with US Census data for the population aged 65 and older in the Boston Metropolitan Statistical Area shown in Table 3, the study sample was generally representative of elders in the Boston area. In part related to study eligibility criteria and geographic boundaries of the recruitment area, study participants had greater educational attainment than elders in the general community (45% and 24% college graduates, respectively).

The study cohort had a high prevalence of obesity (26%) and low levels of physical activity, with 40% reportedly walking less than 1 mile per week (Table 4). In general, participants reported good to excellent health (85%). Moderate to severe depressive symptoms were infrequent, reported by 8% of participants, and 14% reported having a lot of difficulty or inability to walk 1/4 mile (2–3 blocks). Many participants (39%) reported that they fell at least once in the 12 months prior to the baseline interview, and 17% reported falling 2 or more times in the previous year.

**Table 4:** Baseline health characteristics of MOBILIZE Boston Study participants

Health characteristics	N	Percent
Body Mass Index (kg/m2)		
< 21	42	7.2
21–24.9	137	23.4
25–29.9	252	43.0
≥ 30	155	26.4
Self-rated health:		
Good to excellent	510	85.0
Fair or poor	90	15.0
Walking 2–3 blocks:		
No difficulty	433	72.7
Little or some difficulty	82	13.7
A lot of difficulty	46	7.7
Unable to do	35	5.9
Minor or major depression:		
Yes	45	7.5
No	555	92.5
Cognitive function (MMSE):		
18–23	72	12.0
24–28	302	50.3
29–30	226	37.7
Amount of walking per week:		
< 1 mile	243	40.5
1–3.9 miles	153	25.5
≥ 4 miles	180	30.0
missing	24	4.0
Falls in year before baseline:		
None	364	60.7
One fall	131	21.8
≥ 2 falls	101	16.8
missing	4	0.7

### Data collection

The median length of the home interview (timed) was 2:42 hours (inter-quartile range, 2:24 to 3:00 hours). The estimated average length of the clinic exam was 2:45 hours. In general, there was very little missing information in either the home interview or the clinic exam. For example, fewer than 1% of the first 600 participants had missing pain information in the home interviews. All participants performed the 4-meter walk test, and only 2 participants had missing data for the standing balance test. Several participants (16%) were excluded from the leg press testing due to medical conditions such as acute or severe back or leg pain, systolic blood pressure > 200, or recent cataract surgery. An additional 8% were unable to complete the leg press testing because of safety or other concerns by the participant or tester. Compared to participants who completed the leg press testing, those who did not complete these tests were more likely to be older, female, non-white race, with less education, higher BMI, lower MMSE score, poorer self-rated health, and difficulty with walking or stair climbing (data not shown).

Transcranial Doppler testing was completed in 63% of the sample, and partially completed in another 11% of participants. A common barrier to TCD testing is the absence of a suitable temporal window to isonate the middle cerebral artery. Nonetheless, the large number of successfully completed tests is unprecedented in an elderly population-based study and will provide sufficient statistical power for studying the relationship between cerebral blood flow and falls.

No participants reported a serious adverse event as a result of participation in any aspect of the research. In the follow-up calls conducted by research assistants 3 to 5 days following the clinic visit, a few participants reported minor muscle soreness (n = 11), back or joint pain (n = 11), or headaches (n = 3), all of which resolved quickly. All events were reported to the MBS Safety Monitoring Board.

## Discussion

Our results demonstrate the feasibility of a population-based study of community-living persons aged 70 and older to examine a novel set of risk factors for falls. Participants routinely report positive experiences from their participation, resulting in high retention rates. Excellent information has been obtained in the monthly fall calendar postcards that are routinely returned by the majority of participants (approximately 70% fully completed each month), with the remainder contacted by telephone for completion of the calendars at the end of each month. Although several of our methods have not been used in large cohort studies of elders, our findings demonstrate that the lengthy and complex set of study measures have not been overly burdensome to older and frail participants of the MBS. An important element has been the careful attention paid to the comfort and safety of participants. In addition, all study visits with participants have been organized to provide regular breaks and rest periods in order to limit participant burden and fatigue.

Rubenstein recently reviewed several studies describing reasons for falls and found that up to half of falls were attributed to "accidental or environmental" causes [2]. These reports examined many known risk factors such gait and balance limitations, dizziness, vision problems, confusion, and "drop attacks". This later category often included poorly defined problems such as weakness or unexplained loss of stability. The role of cerebral blood flow in such problems has not yet been explored in other fall studies, but are prominent measures in the MBS. Also, the careful assessment of pain and lower extremity impairments in the MBS offers a new opportunity to explain many falls that were previously attributed to vague categories of risk factors. Initially, the MBS research will determine the role of pain, cerebral blood flow, and a number of lower extremity impairments as causes of falls in older persons. Foot disorders will be examined as a possible cause of balance difficulties that could contribute to falling. In addition, serum biochemical abnormalities and genetic polymorphisms can be assessed from the MBS blood samples.

The combined set of assessments of cerebral blood flow, pain, cognitive and physical function, balance and other performance measures, and monthly falls ascertainment, will provide an exceptional new resource for future studies of functional change in the aging population. The availability of DNA will allow for the examination of genetic hypotheses. The rich medication inventory is an important resource for examining questions related to chronic disease prevalence and treatment. A limitation of the database for other prospective studies of falls is our standard but abbreviated set of traditional fall risk factors. Because the program project used a single cohort to study the many aims of 4 projects, it was necessary to limit assessment of fall risk factors that were not a central focus of one or more projects. Thus we chose to abbreviate some of the measures of fall risk factors that were intended only for use as adjustment variables in our multivariable analyses rather than as primary exposure measures. For example, we assessed only distance vision and use of bifocal/multifocal glasses and not specific vision impairments such as visual contrast or depth perception which are aspects of vision associated with falls [98].

Use of the population-based approach in the MBS rather than a more limited clinical sample will allow us to generalize our findings to comparable populations of older adults. Our study cohort is representative of English-speaking older adults living in the community who are without significant cognitive impairment and are able to walk at least short distances in their homes. The latter 2 criteria resulted in a slightly lower proportion of participants aged 85 and older compared to the Census information. However, the primary reason for ineligibility to participate was language. In Boston, the large immigrant population has lower educational attainment than the non-immigrant population [99]. Thus, our English-speaking study participants have higher educational levels than the general Boston population based on the US Census data. Most importantly, from the eligible population, we have enrolled a study cohort at high risk for falls, with comparable fall rates to previous population-based studies [20,100].

## Conclusion

Although previous epidemiologic studies have explored multiple risk factors for falls, the new set of proposed risk factors assessed in depth for this project combined with excellent falls information will advance our understanding of the causes for falls and will lead to new advances in fall prevention. Our results attest to the feasibility of conducting an innovative population-based study of non-traditional risk factors for falls and disability in the older population. Despite the complexity and potential subject burden in this population of seniors, many of whom have activity limitations, our success demonstrates that such studies are feasible and have the potential to yield important findings in previously under-studied areas related to aging. Many participants have expressed enthusiasm for the research and report that they enjoy their participation. The MBS will provide a valuable and extensive new data resource for examining non-traditional risk factors for falls and mobility problems in the older population.

## Competing interests

The authors declare that they have no competing interests.

## Authors' contributions

SGL helped conceive and design the study, contributed to the data analyses, and led the preparation of the manuscript. DPK helped conceive and design the study, directed the study operations, and contributed to the preparation of the manuscript. RNJ helped conceive and design the study, directed the data management and analyses, and contributed to the preparation of the manuscript. AR directed the study sampling and recruitment, helped with the design of the study, reported the initial recruitment results, and contributed to the preparation of the manuscript. MTH contributed to the design and operations of the study, directed the foot disorders study, and contributed to the preparation of the manuscript. FAS Contributed to the design, implementation and analysis of cerebral blood flow studies and manuscript preparation. HGK helped design and supervise the subsensory threshold study and contributed to the manuscript preparation. EJS helped conceive and design the study, helped direct study operations, and contributed to the preparation of the manuscript. MG contributed to all phases of the participant enrollment and study data collection, supervised the day-to-day operations, and contributed to the preparation of the manuscript. MF contributed to all phases of the participant recruitment and enrollment, supervised the day-to-day screening and enrollment, and contributed to the preparation of the manuscript. LAL directed the conceptualization and design of the study, provided oversight to all aspects of the study implementation, and contributed to manuscript preparation. All authors read and approved the final manuscript.

## Pre-publication history

The pre-publication history for this paper can be accessed here:



## References

[B1] Close JC, Lord SL, Menz HB, Sherrington C (2005). What is the role of falls?. Best Practice & Research.

[B2] Rubenstein LZ (2006). Falls in older people: epidemiology, risk factors and strategies for prevention. Age and Ageing.

[B3] Tinetti ME (2003). Clinical practice. Preventing falls in elderly persons. N Engl J Med.

[B4] Wagner EH, LaCroix AZ, Grothaus L, Leveille SG, Hecht JA, Artz K, Odle K, Buchner DM (1994). Preventing disability and falls in older adults: a population-based randomized trial. American Journal of Public Health.

[B5] Ganz DA, Bao Y, Shekelle PG, Rubenstein LZ (2007). Will my patient fall?. JAMA.

[B6] Freedman VA, Hodgson N, Lynn J, Spillman BC, Waidmann T, Wilkinson AM, Wolf DA (2006). Promoting declines in the prevalence of late-life disability: comparisons of three potentially high-impact interventions. The Milbank Quarterly.

[B7] Tinetti ME, Inouye SK, Gill TM, Doucette JT (1995). Shared risk factors for falls, incontinence, and functional dependence. Unifying the approach to geriatric syndromes. JAMA.

[B8] Leveille SG, Bean J, Bandeen-Roche K, Jones R, Hochberg M, Guralnik JM (2002). Musculoskeletal pain and risk for falls in older disabled women living in the community. Journal of the American Geriatrics Society.

[B9] Leveille SG, Ling S, Hochberg MC, Resnick HE, Bandeen-Roche KJ, Won A, Guralnik JM (2001). Widespread musculoskeletal pain and the progression of disability in older disabled women. Annals of Internal Medicine.

[B10] Scudds RJ, Robertson JM (2000). Pain factors associated with physical disability in a sample of community-dwelling senior citizens. J Gerontol A Biol Sci Med Sci.

[B11] Lichtenstein MJ, Dhanda R, Cornell JE, Escalante A, Hazuda HP (1998). Disaggregating pain and its effect on physical functional limitations. The Journals of Gerontology.

[B12] Hossain M (2001). Intra-individual postural blood pressure variability and stroke in elderly nursing home residents. J Clin Epidemiol.

[B13] Kairo K (2001). Lower standing systolic blood pressure as a predictor of falls in the elderly: a community-based prospective study. J Am Coll Cardiol.

[B14] De Reuck J (1971). The human periventricular arterial blood supply and the anatomy of cerebral infractions. Eur Neurol.

[B15] Puisiex F (2001). Relationship between leuko-araiosis and blood pressure variability in the elderly. Eur Neurol.

[B16] Raiha I (1993). Relationship between vascular factors and white matter low attenuation of the brain. Acta Neurol Scand.

[B17] Baloh RW, Ying SH, Jacobson KM (2003). A longitudinal study of gait and balance dysfunction in normal older people. Arch Neurol.

[B18] Starr JM, Leaper SA, Murray AD, Lemmon HA, Staff RT, Deary IJ, Whalley LJ (2003). Brain white matter lesions detected by magnetic resonance [correction of resosnance] imaging are associated with balance and gait speed. Journal of Neurology, Neurosurgery, and Psychiatry.

[B19] Whitman GT, Tang Y, Lin A, Baloh RW (2001). A prospective study of cerebral white matter abnormalities in older people with gait dysfunction. Neurology.

[B20] Tinetti ME, Speechley M, Ginter SF (1988). Risk factors for falls among elderly persons living in the community. N Engl J Med.

[B21] Edelberg HK (2001). Falls and function. How to prevent falls and injuries in patients with impaired mobility. Geriatrics.

[B22] Menz HB, Lord SR (2001). The contribution of foot problems to mobility impairment and falls in community-dwelling older people. Journal of the American Geriatrics Society.

[B23] Menz HB, Morris ME, Lord SR (2006). Foot and ankle risk factors for falls in older people: a prospective study. The Journals of Gerontology.

[B24] Priplata A, Niemi J, Salen M, Harry J, Lipsitz LA, Collins JJ (2002). Noise-enhanced human balance control. Physical Review Letters.

[B25] Priplata AA, Niemi JB, Harry JD, Lipsitz LA, Collins JJ (2003). Vibrating insoles and balance control in elderly people. Lancet.

[B26] Priplata AA, Patritti BL, Niemi JB, Hughes R, Gravelle DC, Lipsitz LA, Veves A, Stein J, Bonato P, Collins JJ (2006). Noise-enhanced balance control in patients with diabetes and patients with stroke. Annals of Neurology.

[B27] Folstein MF, Folstein SE, McHugh PR (1975). "Mini-mental state". A practical method for grading the cognitive state of patients for the clinician. Journal of Psychiatric Research.

[B28] Escobar JI, Burnam A, Karno M, Forsythe A, Landsverk J, Golding JM (1986). Use of the Mini-Mental State Examination (MMSE) in a community population of mixed ethnicity. J Nerv Ment Dis.

[B29] Rose G (1962). The diagnosis of ischaemic heart pain and intermittent claudication in field surveys. Bull WHO.

[B30] Paffenbarger RS, Hyde RT, Hsieh CC, Wing AL (1986). Physical activity, other life-style patterns, cardiovascular disease and longevity. Acta Med Scand Suppl.

[B31] Anderson KO, Dowds BN, Pelletz RE, Edwards WT, Peeters-Asdourian C (1995). Development and initial validation of a scale to measure self-efficacy beliefs in patients with chronic pain. Pain.

[B32] Lorig K, Chastain RL, Ung E, Shoor S, Holman HR (1989). Development and evaluation of a scale to measure perceived self- efficacy in people with arthritis. Arthritis Rheum.

[B33] Glass TA, Mendes de Leon CF, Seeman TE, Berkman LF (1997). Beyond single indicators of social networks: a LISREL analysis of social ties among the elderly. Social Science & Medicine (1982).

[B34] Morisky DE, Green LW, Levine DM (1986). Concurrent and predictive validity of a self-reported measure of medication adherence. Medical Care.

[B35] Katz S, Ford AB, Moskowitz AW, Jackson BA, Jaffe MW (1963). The index of ADL: A standardized measure of biological and psychosocial function. JAMA.

[B36] Lawton MP, Brody EM (1969). Assessment of older people: self-maintaining and instrumental activities of daily living. Gerontologist.

[B37] Rosow I, Breslau N (1966). A Guttman health scale for the aged. J Gerontol.

[B38] Shapiro AM, Benedict RH, Schretlen D, Brandt J (1999). Construct and concurrent validity of the Hopkins Verbal Learning Test-revised. The Clinical Neuropsychologist.

[B39] Benton AL, Hamsher K (1976). Multilingual Aphasia Examination.

[B40] Miceli G, Caltagirone C, Gainotti G, Masullo C, Silveri MC (1981). Neuropsychological correlates of localized cerebral lesions in non-aphasic brain-damaged patients. Journal of Clinical Neuropsychology.

[B41] Perret E (1974). The left frontal lobe of man and the suppression of habitual responses in verbal categorical behaviour. Neuropsychologia.

[B42] Snow WG, Tierney MC, Zorzitto ML, Fisher RH, Reid DW (1988). One year test-retest reliability of selected tests in older adults.. Paper presented at the Annual Meeting of the International Neuropsychological Society, New Orleans.

[B43] Pugh KG, Kiely DK, Milberg WP, Lipsitz LA (2003). Selective impairment of frontal-executive cognitive function in african americans with cardiovascular risk factors. Journal of the American Geriatrics Society.

[B44] Royall DR, Mulroy AR, Chiodo LK, Polk MJ (1999). Clock drawing is sensitive to executive control: a comparison of six methods. J Gerontol B Psychol Sci Soc Sci.

[B45] Royall DR, Cordes JA, Polk M (1998). CLOX: an executive clock drawing task. Journal of Neurology, Neurosurgery, and Psychiatry.

[B46] Shulman KI (2000). Clock-drawing: is it the ideal cognitive screening test?. International journal of geriatric psychiatry.

[B47] Psaty BM, Lee M, Savage PJ, Rutan GH, German PS, Lyles M (1992). Assessing the use of medications in the elderly: methods and initial experience in the Cardiovascular Health Study. The Cardiovascular Health Study Collaborative Research Group. J Clin Epidemiol.

[B48] Herrmann C (1997). International experiences with the Hospital Anxiety and Depression Scale--a review of validation data and clinical results. Journal of Psychosomatic Research.

[B49] Zigmond AS, Snaith RP (1983). The hospital anxiety and depression scale. Acta Psychiatrica Scandinavica.

[B50] Washburn RA, Smith KW, Jette AM, Janney CA (1993). The Physical Activity Scale for the Elderly (PASE): development and evaluation. J Clin Epidemiol.

[B51] Ware J, Kosinski M, Keller SD (1996). A 12-Item Short-Form Health Survey: construction of scales and preliminary tests of reliability and validity. Medical Care.

[B52] Melzack R (1975). The McGill pain Questionnaire:  major properties and scoring methods. Pain.

[B53] Escalante A, Lichtenstein MJ, Lawrence VA, Roberson M, hazuda HP (1996). Where does it hurt?  Stability of recordings of pain location using the McGill Pain Map. The Journal of Rheumatology.

[B54] Cleeland CS, Chapman CR, Loeser JD (1989). Measurement of pain by subjective report. Advances in Pain Research and Therapy.

[B55] Keller SD, Bayliss MS, Ware JE, Hsu MA, Damiano AM, Goss TF (1997). Comparison of responses to SF-36 Health Survey questions with one-week and four-week recall periods. Health Serv Res.

[B56] Tan G, Jensen MP, Thornby JI, Shanti BF (2004). Validation of the Brief Pain Inventory for chronic nonmalignant pain. J Pain.

[B57] Zelman DC, Smith MY, Hoffman D, Edwards L, Reed P, Levine E, Siefeldin R, Dukes E (2004). Acceptable, manageable, and tolerable days: patient daily goals for medication management of persistent pain. J Pain Symptom Manage.

[B58] Hochberg MC, Corti MC, Ferrucci L, Guralnik JM, Guralnik JM, Fried LP, Simonsick EM, Kasper JD, Lafferty ME (1995). Musculoskeletal Disease. The Women's Health and Aging Study: Health and Social Characteristics of Older Women with Disability.

[B59] Cummings SR, Nevitt MC, Kidd S (1988). Forgetting falls. The limited accuracy of recall of falls in the elderly. Journal of the American Geriatrics Society.

[B60] Tinetti ME, Mendes de Leon CF, Doucette JT, Baker DI (1994). Fear of falling and fall-related efficacy in relationship to functioning among community-living elders. Journal of Gerontology.

[B61] Tinetti ME, Richman D, Powell L (1990). Falls efficacy as a measure of fear of falling. Journal of Gerontology.

[B62] Radloff LS (1977). The CES-D Scale:  A self report depresion scale for research in teh general population. App Psych Meas.

[B63] Berkman LF, Berkman CS, Kasl S, Freeman DH, Leo L, Ostfeld AM, Cornoni-Huntley J, Brody JA (1986). Depressive symptoms in relation to physical health and functioning in the elderly. American Journal of Epidemiology.

[B64] Yesavage JA, Brink TL, Rose TL, Lum O, Huang V, Adey M, Leirer VO (1982). Development and validation of a geriatric depression screening scale: a preliminary report. Journal of Psychiatric Research.

[B65] Eaton WW, Muntaner C, Smith C, Tien A, Ybarra M, Maruish ME (2004). Center for Epidemiologic Studies Depression Scale: Review and Revision (CESD and CESD--R). The Use of Psychological Testing for Treatment Planning and Outcomes Assessment.

[B66] Jones RN, Fonda SJ (2004). Use of an IRT-based latent variable model to link different forms of the CES-D from the Health and Retirement Study. Soc Psychiatry Psychiatr Epidemiol.

[B67] Lord FM, Novick MR (1968). Statistical theories of mental test scores.

[B68] Keegan TH, Kelsey JL, King AC, Quesenberry CP, Sidney S (2004). Characteristics of fallers who fracture at the foot, distal forearm, proximal humerus, pelvis, and shaft of the tibia/fibula compared with fallers who do not fracture. American Journal of Epidemiology.

[B69] Koepsell TD, Wolf ME, Buchner DM, Kukull WA, LaCroix AZ, Tencer AF, Frankenfeld CL, Tautvydas M, Larson EB (2004). Footwear style and risk of falls in older adults. Journal of the American Geriatrics Society.

[B70] Altman R, Alarcon G, Appelrouth D, Bloch D, Borenstein D, Brandt K, Brown C, Cooke TD, Daniel W, Feldman D (1991). The American College of Rheumatology criteria for the classification and reporting of osteoarthritis of the hip. Arthritis Rheum.

[B71] Altman R, Alarcon G, Appelrouth D, Bloch D, Borenstein D, Brandt K, Brown C, Cooke TD, Daniel W, Gray R (1990). The American College of Rheumatology criteria for the classification and reporting of osteoarthritis of the hand. Arthritis Rheum.

[B72] Altman R, Asch E, Bloch D, Bole G, Borenstein D, Brandt K, Christy W, Cooke TD, Greenwald R, Hochberg M (1986). Development of criteria for the classification and reporting of osteoarthritis. Classification of osteoarthritis of the knee. Diagnostic and Therapeutic Criteria Committee of the American Rheumatism Association. Arthritis Rheum.

[B73] Wolfe F, Smythe HA, Yunus MB, Bennett RM, Bombatdier C, Goldenberg DL, Tugwell P, Campbell SM, Abeles M, Clark P, al. (1990). The American College of Rheumatology 1990 Criteria for the Classification of Fibromyalgia.  Report of the Multicenter Criteria Committee. Arthritis Rheum.

[B74] Hannan MT, Murabito JM, Felson DT, Rivinus MC, Kaplan J, Kiel DP (2003). The Epidemiology of Foot Disorders and Foot Pain in Men and Women: the Framingham Study.. Arthritis Rheum.

[B75] Hannan MT, Zimmer J, Sullivan E, Kiel DP (2001). Physical Limitations and foot disorders in elders. Journal of the American Geriatrics Society.

[B76] Olaleye D, perkins BA, Bril V (2001). evaluation of three screening tests and risk assessment model for diagnosing peripheral neuropathy in teh diabetes clinic.. Diabetes Res Clin Pract.

[B77] Aaslid R, Markwalder TM, Nornes H (1982). Noninvasive transcranial Doppler ultrasound recording of flow velocity in basal cerebral arteries. Journal of Neurosurgery.

[B78] Babikian V (1999). Transcranial Doppler Ultrasonography.

[B79] Lipsitz LA, Mukai S, Hamner J, Gagnon M, Babikian V (2000). Dynamic regulation of middle cerebral artery blood flow velocity in aging and hypertension. Stroke.

[B80] Mukai S, Lipsitz LA (2002). Orthostatic hypotension. Clin Geriatr Med.

[B81] Sorond FA, Schnyer DM, Serrador JM, Milberg WP, Lipsitz LA (2008). Cerebral Blood Flow Regulation During Cognitive Tasks: Effects of Healthy Aging.. Cortex.

[B82] Berg KO, Maki BE, Williams JI, Holliday PJ, Wood-Dauphinee SL (1992). Clinical and laboratory measures of postural balance in an elderly population. Arch Phys Med Rehabil.

[B83] Shumway-Cook A, Baldwin M, Polissar NL, Gruber W (1997). Predicting the probability for falls in community-dwelling older adults. Physical Therapy.

[B84] Hurvitz EA, Richardson JK, Werner RA, Ruhl AM, Dixon MR (2000). Unipedal stance testing as an indicator of fall risk among older outpatients. Arch Phys Med Rehabil.

[B85] Wood KM, Edwards JD, Clay OJ, Wadley VG, Roenker DL, Ball KK (2005). Sensory and cognitive factors influencing functional ability in older adults. Gerontology.

[B86] Guralnik JM, Simonsick EM, Ferrucci L, Glynn RJ, Berkman LF, Blazer DG, Scherr PA, Wallace RB (1994). A short physical performance battery assessing lower extremity function: association with self-reported disability and prediction of mortality and nursing home admission. J Gerontol.

[B87] Guralnik JM, Ferrucci L, Pieper CF, Leveille SG, Markides KS, Ostir GV, Studenski S, Berkman LF, Wallace RB (2000). Lower extremity function and subsequent disability: consistency across studies, predictive models, and value of gait speed alone compared with the short physical performance battery. The Journals of Gerontology.

[B88] Guralnik JM, Ferrucci L, Simonsick EM, Salive ME, Wallace RB (1995). Lower-extremity function in persons over the age of 70 years as a predictor of subsequent disability. N Engl J Med.

[B89] Borg G (1970). Perceived exertion as an indicator of somatic stress. Scandinavian Journal of Rehabilitation Medicine.

[B90] Kellogg International Work Group on the Prevention of Falls by the Elderly (1987). The prevention of falls in later life. Dan Med Bull.

[B91] Campbell AJ, Borrie MJ, Spears GF (1989). Risk factors for falls in a community-based prospective study of people 70 years and older. J Gerontol.

[B92] Tinetti ME, Liu WL, Claus EB (1993). Predictors and prognosis of inability to get up after falls among elderly persons. JAMA.

[B93] Hale WA, Delaney MJ, Cable T (1993). Accuracy of patient recall and chart documentation of falls. J Am Board Fam Pract.

[B94] Bostrom B, Sandh M, Lundberg D, Fridlund B (2003). A comparison of pain and health-related quality of life between two groups of cancer patients with differing average levels of pain. J Clin Nurs.

[B95] Saal JA, Saal JS (2000). Intradiscal electrothermal treatment for chronic discogenic low back pain: a prospective outcome study with minimum 1-year follow-up. Spine.

[B96] Hougaard P (2000). Analysis of Multivariate Survival Data.

[B97] Therneau T, Grambsch PM (2000). Modeling Survival Data : Extending the Cox Model.

[B98] Lord SR, Dayhew J (2001). Visual risk factors for falls in older people. Journal of the American Geriatrics Society.

[B99] Jimenez CR (2006). New Bostonians Demographic Report.

[B100] Lord SR, Ward JA, Williams P, Anstey KJ (1994). Physiological factors associated with falls in older community-dwelling women. Journal of the American Geriatrics Society.

